# Prophylactic Pulmonary Artery Banding in Pediatric Dilated Cardiomyopathy: An Additional Therapeutic Option

**DOI:** 10.3390/jcdd11030079

**Published:** 2024-02-27

**Authors:** Elena Panaioli, Diala Khraiche, Margaux Pontailler, Flavie Ader, Olivier Raisky, Regis Gaudin, Damien Bonnet

**Affiliations:** 1 Unité Médico-Chirurgicale de Cardiologie Congénitale et Pédiatrique, Centre de Référence Malformations Cardiaques Congénitales Complexes—M3C, Hôpital Universitaire Necker-Enfant-Malades, 75015 Paris, France; epanaioli@gmail.com (E.P.); margaux.pontailler@aphp.fr (M.P.); olivier.raisky@aphp.fr (O.R.); regis.gaudin@aphp.fr (R.G.); damien.bonnet1@gmail.com (D.B.); 2 Service de Biochimie Métabolique, UFCardiogénétique et Myogénétique, Département Médico-Universitaire BioGEM, APHP, Hôpital Universitaire Pitié-Salpêtrière, 75013 Paris, France; flavie.ader@aphp.fr; 3 M3C-Necker, Hôpital Universitaire Necker-Enfants Malades, AP-HP, Université Paris Cité, 75006 Paris, France

**Keywords:** dilated cardiomyopathy, heart failure, pulmonary artery banding, toddlers, ventricular aneurysm

## Abstract

Dilated cardiomyopathy (DCM) is the most common childhood cardiomyopathy and is associated with considerable early mortality. Heart transplantation is often the only viable life-saving option. Pulmonary artery banding (PAB) has been recently proposed as a bridge or alternative to transplantation for DCM. In our cohort, PAB was selectively addressed to heritable DCM or DCM with congenital left ventricle aneurysm (CLVA). This study aimed to describe the clinical evolution and left ventricle reverse remodeling (LVRR) over time (6 months and 1 year after surgery). Ten patients with severe DCM received PAB between 2016 and 2021 and underwent clinical and postoperative echocardiography follow-ups. The median age at PAB was <1 year. The in-hospital mortality was zero. Two patients died two months after PAB of end-stage heart failure. The modified Ross class was improved in the eight survivors with DCM and remained stable in the two patients with CLVA. We observed a positive LVRR (LV end-diastolic diameter Z-score: 8.4 ± 3.7 vs. 2.8 ± 3; *p* < 0.05; LV ejection fraction: 23.8 ± 5.8 to 44.5 ± 13.1 (*p* < 0.05)). PAB might be useful as part of the armamentarium available in infants and toddlers with severe DCM not sufficiently responding to medical treatment with limited probability of spontaneous recovery.

## 1. Introduction

The incidence of dilated cardiomyopathy (DCM) in toddlers is 8.34 cases per 100,000 per year, and it is still a leading cause of death [1,2]. The prognosis depends on age, heart failure status, and etiology [2]. DCM of the left ventricle (LV) is characterized by a reduction in the LV ejection fraction (LVEF) below 50% and a dilatation of the LV with Z-score values above +2 of the LV end-diastolic dimensions. Severe LV dilatation, low LV ejection fraction, and mitral regurgitation have been associated with a poor outcome in DCM [3]. The ultimate therapy is heart transplantation. However, there is limited availability of heart transplantation in infants, as well as worrying long-term outcomes. Alternative therapeutic strategies should be considered to at least delay listing for transplantation or to avoid mechanical ventricular support for children on the waiting list [4]. Pulmonary artery banding (PAB) is an old technique that was initially applied to balance the systemic–pulmonary circulations in cases of ventricular shunts [5]. It has more recently been used in patients with congenitally corrected transposition of the great arteries or after the atrial switch operation for transposition of the great arteries for retraining the sub-pulmonary LV after a “double switch” procedure [6]. Our group has promoted PAB in infants with double discordance to maintain the geometry of the two ventricles, to limit the occurrence of severe tricuspid regurgitation, and to preserve the option of performing a double switch procedure later in life without the need to retrain the left ventricle [7]. Based on these different examples, as well as the ventricle–ventricular interactions, Schranz et al. [8] proposed an innovative application of the PAB to palliate end-stage heart failure due to DCM in young children (<6 years of age) with preserved right ventricular function. Spigel et al. completed a pioneer study considering PAB in DCM and critical clinical conditions requiring non-invasive ventilation and inotropic support [9]. However, an open debate is still ongoing about the indications of PAB in pediatric DCM. Whether this therapy is a bridge to cardiac transplantation or a destination therapy also remains to be evaluated. Here, we report the result of the effect of PAB in infants with DCM and severe heart failure not responding to maximal medical therapy. In this study, PAB has been selectively addressed to children with heritable DCM or DCM associated with congenital left ventricular aneurysm (CLVA) assuming that the presence of CLVA implies a worse prognosis [10].

## 2. Materials and Methods

### 2.1. Cohort, Demographic, and Clinical Data

This is a retrospective review of data collected prospectively in the context of usual care. According to the standard practice of our department, clinical information, in-hospital clinical outcomes, and procedure details of all our patients are prospectively entered into a dedicated database. In our institution, ten patients with severe DCM received a surgical PAB between 2016 and 2021. All patients in our study population received the maximal medical treatment according to current recommendations [11]. According to the previous results of PAB in DCM, our patients were not eligible for PAB if they were older than 2 years or if they presented right ventricular failure with tricuspid regurgitation (≥moderate). As far genetic variants are concerned, they are classified into 5 classes: certainly pathogenic variants for already-described variants (P, class 5), likely pathogenic variants for new variants presenting all pathogenicity criteria (LP, class 4), and variant of unknown significance (VUS, class 3). We have considered PAB in children with LV DCM carrying a variant class of 4 or 5 in the known Mendelian disease genes causing cardiomyopathies or severe LV systolic dysfunction associated with CLVA. This retrospective study was approved and recorded in the digital data processing registry of the APHP (Assistance Publique-Hôpitaux de Paris, protocol registration number: 2022 0722163157).

We collected demographic, clinical, and echocardiographic data prior to surgery, at 6 months and 1 year after PAB. The modified Ross class [12] was used to evaluate the clinical status of each patient. Plasma B-type natriuretic peptide (BNP) or amino-terminal pro-peptide counterpart (NT-proBNP) values were collected as routine laboratory monitoring. We considered NT-proBNP normal values as < 379.7 ng/L and <362.6 ng/L in males and females, respectively [13]. BNP value < 100 pg/mL was considered normal.

### 2.2. Cardiac Imaging

All patients underwent a baseline echocardiography before PAB, and echocardiographic follow-ups at 6 months and 1 year after PAB. DCM was diagnosed according to the following criteria: dilation of the LV (Z-score values > +2) associated with LVEF < 50%. The echocardiographic examination followed a standardized protocol on a Vivid E9 device (General Electric Vingmed Ultrasound, Horten, Norway). LV end-diastolic internal diameter was measured at end diastole with M-mode echocardiography at midventricular level. According to guidelines from the American Society of Echocardiography (ASE), the biplane method of discs for ejection fraction (EF) quantification is preferred [14,15]. These measurements were expressed as Z-score values adjusted for body surface area as compared with the normal pediatric population [16,17]. For deformation analysis, endocardial borders were traced at the end-diastolic frame in apical views. Peak longitudinal strain was computed automatically, generating regional data from 6 segments and an average value for each view to obtain the mean value of global longitudinal strain (GLS). Peak average longitudinal strain was measured in the apical 4-chamber and apical 2-chamber views (in 6 segments from each view) and considered in our retrospective analysis [18,19,20]. Right ventricle (RV) function was evaluated with TAPSE and RV fractional area change [14]. Finally, CLVA was defined by the presence in CMR and/or echocardiography of an akynetic or dyskinetic ventricular outpouching with a wide connection to the ventricle. Cardiac magnetic resonance imaging (CMR; 1.5 Tesla, GE, General Electrics) was performed before PAB in 4/8 patients (2 with DCM and 2 with CLVA), and one year after PAB, in one patient with CLVA, cardiac magnetic resonance imaging was performed using a 1.5 Tesla magnet (MR450 GE Medical systems, Milwaukee, WI, USA). Image acquisition was conducted with a 32-channel phased-array cardiac coil and a vector electrocardiogram for R-wave triggering using a standard MRI imaging protocol. The images were acquired during a breath hold. LV dimensions and function were obtained from a steady-state free precession short-axis stack (FIESTA), as well as 2- and 4-chamber views. This was followed by the administration of gadolinium-based contrast agents with standard inversion-recovery gradient-echocardiographic imaging performed at 10 to 15 min in matched imaging views. Myocardial fibrosis on LGE imaging was considered present when extending beyond ventricular insertion areas, and its corresponding patterns were coded based on visual assessment.

### 2.3. Procedural Data

**Figure 1:**
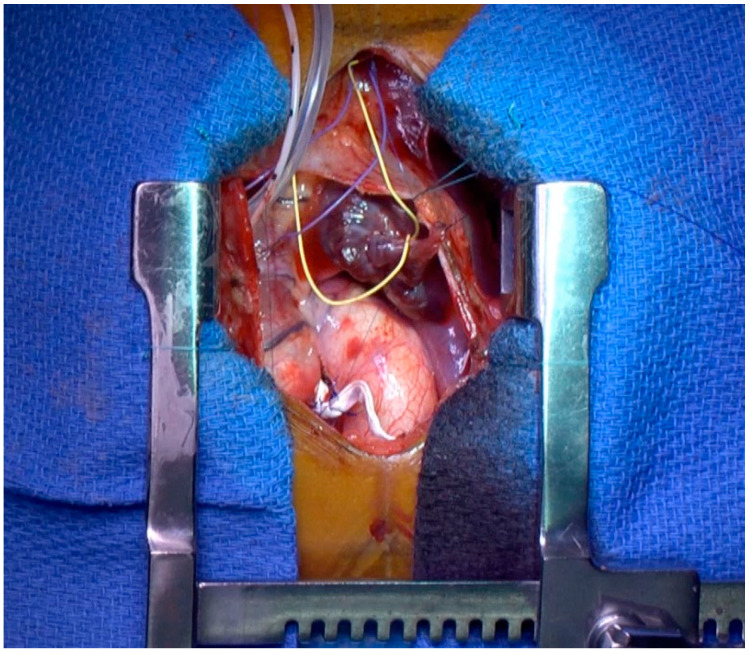
Surgical view of RV catheter for RV measurement and PAB (white) at the level of pulmonary artery.

The surgical technique for PA banding and the perioperative hemodynamics objectives have already been largely described [8,9,10]. Briefly, surgery was performed under direct pressure monitoring (arterial, right atrial, and RV pressures) and continuous trans-esophageal echocardiographic guidance (Figure 1). The main pulmonary trunk was exposed through a midline sternotomy and upper pericardiotomy. The PAB was tailored from a 0.4 mm polytetrafluoroethylene (PTFE) patch to obtain a width of 3 mm. The band was marked to have a circumferential length in the range of 24 + 1/kg mm. The band was progressively tightened, with the optimal degree of constriction being achieved when the RV pressure was approximately 60–70% of the systemic level with no echocardiographic signs of significant tricuspid regurgitation or RV dilatation and/or failure and septal configuration improvement with a shift towards the midline position. Finally, the PAB was secured, and the pericardium was closed.

### 2.4. Statistical Analysis

The statistical analysis was performed with a commercially available package (SPSS, Rel 18.0 2009, SPSS Inc., Chicago, IN, USA). Categorical variables were expressed as percentages. We used the Shapiro–Wilk test to evaluate the normality of the distribution of results. The continuous variables having a normal distribution were expressed as mean values, accompanied by their standard deviation. All continuous variables having a non-normal distribution were expressed as median values and 1st and 3rd IQR range. Analysis of continuous variables was performed with the use of a paired t-test rejected for a *p* < 0.05. Categorical variables were analyzed with a two-tailed Fischer’s exact test.

## 3. Results

### 3.1. Study Population at Diagnosis and Clinical Evolution before PAB

The cohort consisted of five girls and five boys with a median age at diagnosis of 2.4 months (range: 0.1–12.6 months). Table 1 describes the etiology of heart failure and clinical status at diagnosis.

One patient was a composite heterozygote with two variants in the CSRP3 gene and in the MYH6 gene. For CLVAs, the localization for CVLA1, 2, and 3 was at the lateral wall of the LV, midventricular–apical interventricular septal segments, and apical segments of the LV, respectively. 

Five of the seven (71.4%) DCM patients received DCM diagnosis after intensive care unit (ICU) admission for cardiogenic shock. All of them received inotropic support with milrinone during their ICU stay. The DCM2 patient consulted because of failure to thrive (modified Ross class 3). The DCM3 patient’s diagnosis was conducted during familial screening for DCM. The latter was in modified Ross class 1 at the time of diagnosis but with rapid pejorative evolution. The three patients with CLVA were in modified Ross class 1 at the time of diagnosis (Table 1).

At discharge from the ICU or hospital, all patients received ACE inhibitors and betablockers as a standard of care protocol in our department. In addition, all patients with DCM and one patient with CLVA received mineralocorticoid receptor antagonists. Six out of ten patients received loop diuretics.

### 3.2. Procedural Data and Periprocedural Outcome

For all patients, the heart transplantation’s indication was discussed with the pediatric “Heart failure team”. PAB was considered a reasonable approach for the patients who were not improving with standard medical care. A complete pre-transplantation work-up was performed, and the parents’ informed consent for PAB was obtained. No patient was in the cardiac ICU at the time of PAB.

After an individualized tightening of the PAB, a systolic RV pressure at a level of 60% to 70% of the systolic aortic blood pressure was achieved in all patients. In one patient, a dual-chamber pacemaker was implanted due to the occurrence of a complete AV block during the procedure, while a left bundle branch block had been diagnosed before surgery. One patient had a severe mitral valve regurgitation at the time of PAB because of annulus dilatation. The patient was symptomatic of this volume overload, and we decided to perform a partial annuloplasty. The reparation consisted of a mitral annuloplasty at the same time as PAB. There were no other mechanisms of mitral regurgitation besides annulus dilatation. 

PAB was successfully performed in all patients with no in-hospital mortality. The mean ICU stay was 7.5 ± 3.7 days. The mean duration time of standard postoperative inotropic support was 5.1 ± 2.6 days. The mean duration of invasive ventilatory support was 3.5 ± 2.1 days, and two patients needed non-invasive ventilation for a period of two additional days after extubation. Two patients required treatment with levosimendan before ICU discharge for clinical and echocardiographic worsening. One patient experienced episodes of atrial tachycardia treated with betablockers. 

At hospital discharge, the mean value of the Doppler flow velocity across the PAB was 3.1 ± 0.6 m/s.

### 3.3. Clinical Status and Reversal Ventricular Remodeling from PAB to 1 Year of Follow-Up

**Figure 2:**
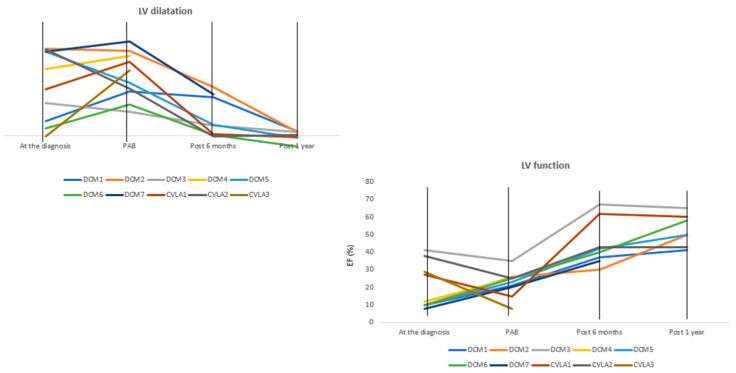
Left ventricle parameters at diagnosis and at PAB and evolution of left ventricle reverse remodeling over time after 6 months and 1 year.

At the time of PAB, 5/10 children were in modified Ross class 3 with persisting symptoms including failure to thrive, congestion signs, and lack of improvement in LV function. These symptoms were associated with intermittent and recurrent signs of low cardiac output in the majority of them. Hospitalization for an episode of heart failure occurred in three patients within 3 months prior to PAB. The median value of NT-proBNP was 2860 pg/mL (IQR1 365.5–IQR3 5772.5 pg/mL). Figure 2 outlines that there was no significant improvement with medical therapy.

Table 2 summarizes the evolution of vital and clinical status and heart failure therapy from PAB to 1 year of follow-up. For DCM3, the data at 1 year were collected later than at 1 year due to pandemic lockdown. This patient underwent a dilatation of the PAB at 16.6 months because the right ventricle was hypokinetic with isosystemic RV pressure. Two out of ten patients died after PAB because of worsening heart failure. The first patient died 69 days after PAB of severe heart failure associated with RSV bronchiolitis. The second patient died 77 days after PAB after having been admitted to the ICU for a low cardiac output condition. The mean time of follow-up was 2.1 years ± 1.7. Overall, the modified Ross class improved in all survivors with DCM and remained stable in the two patients with CLVA.

Growth was improved with a significant increase in the weight–length Z-score from discharge to 6-month follow-up (−0.9 ± 1.4 vs. 0.4 ± 1.1; *p* 0.02) that remained stable thereafter. Heart failure therapy included ACE inhibitors, betablockers, and mineralocorticoid receptor antagonists in the majority of patients with only one still receiving loop diuretics at one-year follow-up. None of the survivors had been readmitted for worsening heart failure during follow-up. When available, NT-proBNP/BNP values decreased after the PAB. More precisely, in DCM1, DCM3, DCM5, and DCM7, there was a reduction of 77%, 89%, 95%, and 98%, respectively, at 6 months. In CLVA patients, the values of NT-proBNP stayed stable at the available controls.

At the time of PAB, LV systolic function was severely reduced (LVEF 22.3 ± 7.2%,GLS −8.1 ± 2.5%) and the LV was severely dilated with a Z-score value > +5 (Z-score value 8.9 ± 3.5), despite optimal medical treatment. Mitral regurgitation was severe in one out of ten patients; the remaining nine patients had mild to moderate mitral regurgitation. The RV systolic function was normal in all patients.

CMR before PAB was available in four out of seven DCM patients (57%) and showed no myocardial fibrosis. A significant progressive reduction in the left heart chambers’ dilatation and improvement in LV function was observed compared to baseline values (see Videos S1 and S2). We found a significant statistical difference in the LVEDD Z-score from the time of PAB (8.4 ± 3.7) to 6 months (2.8 ± 3, *p* 0.03) with a persistent reduction at 1 year (0.01 ±0.8, *p* 0.02). We observed a similar trend for the EF, with an improvement in the systolic function at PAB (23.8 ± 5.8) compared to at 6 months (44.5 ± 13.1, *p* 0.01). At one year, the increase in the EF persisted (52.4 ± 4.9, *p* 0.00).

Moreover, the GLS (%) significantly increased from the time of PAB (−8.2 ± 2.4) to at 6 months (-11.8 ± 3.3, *p* 0.04) and 1 year (−13.5 ± 1.9, *p* 0.01).

**Figure 3:**
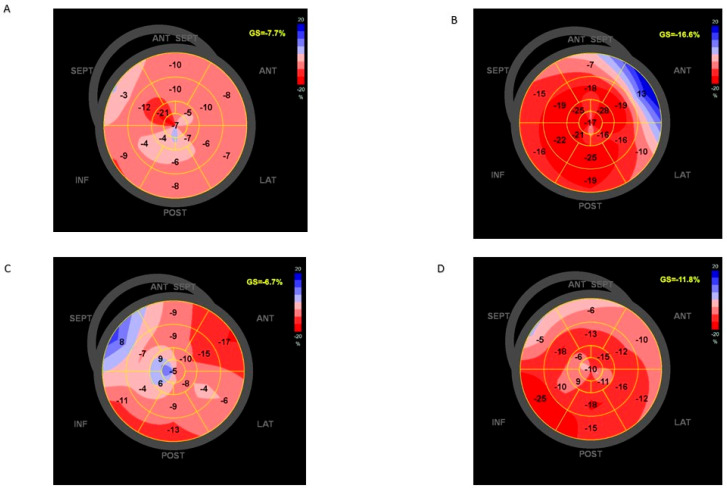
(**A**) GLS of DCM patient before PAB; (**B**) GLS of DCM patient after PAB; (**C**) GLS of CLVA patient before PAB; (**D**) GLS of CLVA patient after PAB. GLS, global longitudinal strain; DCM; dilated cardiomyopathy; CLVA: congenital left ventricle aneurysm; PAB: pulmonary artery banding.

Figure 3 shows the improvement in the GLS in DCM and DCM associated with CLVA after PAB. 

Finally, we observed a progressive dilatation of the RV from the time of PAB (6.1 ± 1.7) to 6 months (8.2 + 1.8, *p* 0.004) and a mild reduction in the systolic function estimated with the RVFAC (Table 2).

The LVEF in patient DCM4 was 15% at discharge, with severe dilatation of the LV and mild mitral regurgitation. The Doppler flow velocity was 3 m/s across the PAB. During follow-up, the LVEF improved to reach 30% at 6 weeks post-PAB, but concomitantly, the mitral regurgitation worsened, and the left heart chambers were considerably dilated (indexed LV end-diastolic volume 225 mL/m^2^; indexed left atrium volume 140 mL/m^2^). RV systolic function was preserved, and the Doppler flow velocity across the PAB was 2.3 m/s. After discharge, the patient was followed in the day hospital care unit with progressive therapy optimization as re-introduction and an increase in betablockers. We observed a transient clinical improvement with a reduction in congestive signs. Finally, the child was admitted with end-stage heart failure on day 56 with viral pneumonia. The patient DCM4 was listed for heart transplant, but the parents refused a temporary mechanical circulatory support device. The patient was assisted with ECMO and with an ASD creation with a hybrid procedure in order to reduce the volume overload clinical signs in the context of pneumo-pneumonia. The patient died after 12 days on mechanical support. The patient CLVA3 was hospitalized due to the rapid worsening of heart failure with low-output cardiac signs. More precisely, after an uneventful course after PAB, the LVEF was 30% at discharge with a Doppler flow velocity of 2.5 m/s across the PAB. During follow-up, the LVEF did not improve (25%) and the gradient across the PAB remained stable, but the mitral regurgitation increased. The child was readmitted for worsening heart failure. The therapeutic project was discussed with the parents and medical staff: the collegial decision was to set up comfort care and refuse further invasive procedures. There was no further medical escalation, leading to death 2.5 months after PAB. The latter led to RV remodeling as expected. The RV systolic function estimated with RVFAC decreased after 6 months with no further deterioration at one year, keeping in mind that after this delay, as shown in Table 2, some patients of this series required PAB dilatation of RV failure related to suprasystemic RV systolic pressure. Indeed, dilatation of the PAB was performed at 59.8, 23.9, and 16.6 months after PAB in three survivors.

## 4. Discussion

Surgical PAB is now becoming a more integrated part of the therapeutic arsenal for the treatment of pediatric DCM, whether for cardiac recovery and FR or bridging to transplantation [8,9,20,21,22]. In our study, we have demonstrated a positive reverse remodeling after PAB in genetic DCM and CVLA. Moreover, in our cohort, the PAB was not considered in life-saving conditions but as an additional preventative treatment as part of the medical strategy: none of our patients were in a pediMACS 1, 2, or 3 class at the time of PAB. In our small series, the early mortality was null and without prolonged inotropic or ventilator support during ICU stays. After discharge, within three months, two patients died. The one-year follow-up shows sustained clinical improvement in the survivors with no hospitalization for heart failure after discharge. This was associated with favorable remodeling of the left ventricle in all patients, a reduction in heart failure therapy, and a decrease in the B-natriuretic peptides from the baseline.

The mechanical and functional interdependence between the RV and LV is the fundamental element to understanding the beneficial effect of PAB in LV dilatation and dysfunction. In animal PAB models, increased RV afterload leads not only to RV but also to LV reduced contractility, with myocyte hypertrophy and fibrosis. In these models, not only septal shift but also septal configuration modifies regional wall shear stress. A slight increase in the LV afterload and angiotensin receptor blockade improve LV as well as RV contractility in the same models [23,24]. Whether this strategy will increase LV contractility through shared myocardial fibers has not been shown [7,24]. Two out of ten patients died in our series. This mortality is comparable to that of infants < 10 kgs on the waiting list for heart transplantation. This might be due to an inadequate degree of PAB, but the Doppler flow velocity across the banding was similar to that of the survivors. A difficult evaluation of RV function might also play a role. Finally, both had worsening mitral regurgitation, and more caution in the analysis of the mechanism of mitral regurgitation could help in the selection of responders and non-responders to PAB in this setting. 

The study population of the previously reported series is heterogeneous in terms of the cause of left heart failure, and the diagnostic work-up does not always exclude reversible forms of DCM such as acute myocarditis [20,21,22,25,26]. Some patients in relatively stable condition were also included in these reports. In our center, we selected patients who were worsening or not improving with maximal heart failure therapy and in whom heart transplantation or VAD assist device implantation were discussed. While some DCM may have a favorable evolution with therapy in young children, death or transplantation occurred in 26% of patients with childhood DCM within 1 year of diagnosis and in ~1% per year thereafter [26,27,28]. In this series, carriers of class 5/class 4 variants in DCM genes were submitted to PAB as we know that they might have a poorer prognosis and a reduced rate of recovery [28]. Moreover, the Rikada study showed that the detection rate of more than one genetic variant of interest was higher in heart transplant recipients compared to non-transplanted patients [28], suggesting an effect of genetic load in DCM severity. Our study is also original because it describes for the first time the results of PAB in children with CLVA, a condition considered to have a poorer prognosis [10]. Infants with CLVA rapidly showed an adverse remodeling with rapid progressive LV dilatation and worsening of the LVEF. The two survivors with CLVA had a preserved functional status with no significant worsening during follow-up and are still alive more than 1 year after PAB. Finally, the aneurysm localization in CLVA might be an important predictor of efficacy in this particular setting, as the CVLA3 patient presenting an apical aneurysm died. This peculiar localization might have influenced the probability of efficacy of the PAB with regard to the shift of the septum and the remodeling of the LV.

Time is certainly an important factor in this strategy. First, the biological crosstalk between the two ventricles necessitates sufficient time to allow for adequate hemodynamic stabilization [23]. Moreover, one of the two patients was older at the time of surgery (23.8 months), and the risk–benefit of PAB seems to be inversely related to age, particularly in severe DCM [23]. Indeed, the regenerative properties of myocardium decrease rapidly after birth, and this might be one of the reasons for PAB failure in the older patient in whom a reduced cardiac regenerative potential could be hypothesized [29]. While the LV diameter almost normalized in survivors after 6 months with concomitant and sustained improvement in the LVEF, the long-term effect of PAB on the RV and consequently on the LV function remains unknown. It is however of note that the GLS increased significantly during the one-year follow-up, coherently with the analysis of the biventricular response to PAB published by Latus et al., where the increase in LV strain indices was associated with an improvement in intraventricular and interventricular synchrony [30]. Finally, we observed a reduction in left atrium dilatation with a Z-score value below <2 already at 6 months. Goldberg et al. have demonstrated that a progressive reduction in left atrial pressure was observed during the gradual increase in the tightening of PAB [31]. Therefore, it is reasonable to suggest that LV preload reduction is an immediate effect of this procedure, inducing a reduction in LV preload and changes in the end-diastolic/end-systolic filling pressures. While the results of this small series are encouraging, the small number of patients and the retrospective design limit the conclusions. Precise sequential and multimodality follow-up was not available for all patients, as some were living overseas in remote French territories. We did not have a control group of children with DCM in whom we could describe adverse or favorable LV remodeling with heart failure therapy alone. Beyond this pilot study, we would like to explore more precisely the mechanisms of action of PAB on ventricular function and interaction. We also aim to clarify the place of PAB in the therapeutic strategy for heart failure in infants and toddlers. Other issues are important in the near future, such as the optimal degree of PAB, the evolution of the RV function with a potential deleterious effect on LV function in the long term, and the time/method of PAB dilatation.

## 5. Conclusions

PAB might be proposed as part of the armamentarium available in infants and toddlers with severe LV dysfunction not sufficiently and spontaneously responding to medical treatment in terms of LV dilation, persistence of MR, and low EF. We found a significant improvement in the clinical status and biological markers associated with left ventricle positive remodeling in 8/10 toddlers. The long-term outcomes have to be further evaluated. Whether this method is only a bridge to heart transplantation or a destination therapy in selected cases has to be demonstrated by larger studies and long-term follow-up of these children.

## Figures and Tables

**Table 1:** Etiology of heart failure and clinical status at diagnosis. DCM: dilated cardiomyopathy; CLVA: congenital left ventricular aneurysm; P/LP: pathogen/like pathogenic; ICU: intensive care unit.

	Heart Failure Etiology			Clinical Status at Diagnosis			
	P/LP Variant 1	P/LP Variant 2	Genetic Testing of Parents	Age (Months)	Cardiogenic Shock/ICU	Modified Ross Class	Weight–Length (Z-Score)
DCM1	ACTC1 c664G>A (p.(Ala222Thr)); class 4	None	None	0.7	Yes	4	−2.7
DCM2	VCL c.1225C>T(p.(Arg409)); class 4	None	VCLc.1225C>T(p.(Arg409)) (mother)	6.1	No	3	−3.60
DCM3	FLNC c.3937C>T(p.(Arg1313)); class 4	None	Not available	2.3	No	1	−1.4
DCM4	CSRP3 c140C>G (p.Thr47Arg); class 4	MYH6 c4999G>A (p.Asp1667Asn); class 4	MYH6 c4999G>A (p.Asp1667Asn) (mother); CSRP3 c140C>G (p.Thr47Arg) (father)	1.9	Yes	4	−1.4
DCM5	DTNA c1841G>A (p.(Arg614Lys); class 3	None	Not available	8.9	Yes	4	−0.4
DCM6	ALMS1. c.2816T>A,pLeu939 *; class 5	ALMS1. c.2816T>A,pLeu939 *	ALMS1; c.2816T>A,pLeu939 * (mother, father)	0.8	Yes	4	−1.87
DCM7	FLNC phe1529leufs10; class 5	None	Not available	4.96	Yes	3	−0.11
CLVA1				12.6	No	1	+1.2
CLVA2				1.9	No	2	−0.5
CLVA3				0.10	No	1	−1.20

* non-sense mutation leading to a premature stop codon.

**Table 2:** Patients’ characteristics at the time of PAB and during follow-up; 6 m/1 y, six-month and one-year follow-up; DCM, dilated cardiomyopathy; CLVA, congenital left ventricular aneurysm; PAB, pulmonary artery banding; LVEDD: left ventricular end-diastolic diameter; EF: ejection fraction; LAD: left atrium diameter; RVFAC: right ventricular factional area change; RVED: right ventricular end diastolic.

	Clinical Data at Time of PAB and During Follow-Up (6 Months, 1 Year)						Echocardiographic Data at Time of PAB and During Follow-Up (6 Months, 1 Year)				
	Weight–Length (Z-Score)	Modified Ross Class a	NT-proBNP (pg/mL)	Enteral Nutritio n	Loop Diuretic	Status at 6 m/1 y	LVEDD Z-Score	EF (%)	LAD Z-Score	RVFAC (%)	RVED Surface
DCM1	−1.3/−0.9/−0.73	3/2/2	2663/605/-	Yes/No/No	Yes/Yes/Yes	Alive	6.5/5.8/0.73	21/37/41	4.5/2.7//1.1	63/55/41	6.5/9.3/9.1
DCM2	−0.51/−0.6/−0,56	3/3/2	2080/-/-	Yes/No	Yes/Yes/No	Alive	12.6/7.3/0.6	26/30/50	4.5/1.4/1.3	49/45/-	4/7.2/-
DCM3	−1.8/−0.5/−0.9	2/2/2	422/43/183	Yes/No/No	N/No/No	Alive	3.6/1.5/0.6	35/67/65	−0.2/-/−0.9/−1.6	52/52/27	7/11/17
DCM4	+1.3/-/-	3-/-	3980/-/-	Yes/-/-	Yes/-/-	Decease d (day 69 afterPAB)	11.8/-/-	25/-/-	6/-/-	44/-/-	8.7/-/-
DCM5	+0.3/+0.5/+0.2	2/2/2	576/202/324	Yes/No/No	Yes/No/No	Alive	7.9/1.7/−0.3	23/42/50	3.3/0.1/0.5	53/40/40	6.5/8.8/8.3
DCM6	+0.8/+2.3/2.02	3/2/2	101/-/-	Yes/No/No	Yes/No/No	Alive	4.6/0.2	25/40	3.6/1/-	56/44/-	4/5/-
DCM7	−2.5/+1.2	3/2/2	16,200/303/-	Yes/No/No	Yes/No/No	Alive	14/6.2/5.4	20/35/32	2.4/1.4/2.5	30/45/-	6.5/7.5/-
CLVA1	+0.4/+1.3/+1.2	1/1/1	576/611,6	No/No/No	No/No/No	Alive	11/0.24/−0.2	15/43/60	1.1/−0.3/-	60/51/-	9/9.4/-
CLVA2	−2.9/−0.4/+0.4	1/1/1	196/-/180	No/No/No	No/No/No	Alive	7.1/−0.1/0.2	25/43/43	0.3/−1.1/−3	47/43/42	5/7.6/8
CLVA 3	−0.04/-/-	3-/-	11,000/-/-	No/-/-	Yes/-/-	Deceased (day 77 after PAB)	9.6/-/-	8/-/-	4.9/-/-	48/-/-	5.5/-/-

## Data Availability

All data are available on the database “Carpedem” on the website Necker’s Hospital.

## Supplementary Materials

The following supporting information can be downloaded at: https://www.mdpi.com/article/10.3390/jcdd11030079/s1, Video S1: The video shows the left chambers’ remodeling and LV systolic function improvement 6 months after PAB in a patient with DCM. Video S2: The video shows the left chambers’ remodeling and LV systolic function improvement 6 months after PAB in a patient with CLVA.
